# Serodiscordance predictors among couples in the HIV context: implications for health care

**DOI:** 10.1186/s12889-021-11835-0

**Published:** 2021-10-13

**Authors:** Marcela Antonini, Priscila Silva Pontes, Elizabete Santos Melo, Regina de Souza Alves, Elucir Gir, William Sorensen, Renata Karina Reis

**Affiliations:** 1 Department of General and Specialized Nursing, University of São Paulo, Ribeirão Preto College of Nursing, Bandeirantes Ave, 3900, Vila Monte Alegre SP, CEP, Ribeirão Preto, 14040-902 Brazil; 2 Paulista University at São José do Rio Preto, São José do Rio Preto, São Paulo Brazil; 3 Department of Health and Kinesiology, University of Texas at Tyler, Tyler, TX USA

**Keywords:** HIV infections / transmission, HIV infections / prevention & control, Sexual behavior, Counseling, Sexual health, Sexual partners, Serodiscordant couples, Health personnel, Health care quality, Access, and evaluation

## Abstract

**Background:**

After HIV diagnosis, people maintain, reestablish their sexual lives, or build new relationships, often with HIV seronegative partners. Therefore, understanding the factors concerning couple-vulnerability is essential in order to design effective HIV preventive strategies. We examined HIV serodiscordant couples prevalence and their associated factors from a Brazilian city.

**Methods:**

This is a cross-sectional analytical study carried out with people living with HIV (PLHIV) who had an active sex life and were engagement in HIV health care follow-up. Data were collected using a semi-structured questionnaire during individual interviews. We analyzed data using bivariate and multiple logistic regression analyses.

**Results:**

There was 72.0% of HIV serodiscordant partnerships. Those who inconsistently used condoms (aOR: 0.3[0.13–0.7]) and/or had HIV detectable viral load (aOR: 0.29 [0.12–0.7]) were less likely to have an HIV serodiscordant sexual partner. On other hand, the lack of HIV transmission counseling by the health service (aOR: 5.08 [2.02–12.76]), or those who had a casual partner (aOR: 8.12 [1.7–38.8]) or a steady and casual one concomitantly (aOR: 24.82 [1.46–420.83]), were more likely to indicate an HIV serodiscordant partnership.

**Conclusion:**

The findings showed a high prevalence of serodiscordant partnerships in PLHIV. Greater visibility among couples in the health services is needed as well as a reassessment in order to provide PLHIV and their sexual partners with care strategies, by the health professionals.

**Supplementary Information:**

The online version contains supplementary material available at 10.1186/s12889-021-11835-0.

## Background

Sexual transmission of the human immunodeficiency virus (HIV) remains the predominant form of HIV transmission in the world [1]. According to the Joint United Nations Program on HIV / AIDS (Unaids) [1], 1.7 million new infections were registered in the world in 2019, with about 62% belonging to adults considered as key populations (gay men and other men who have sex with men, sex workers, people who use drugs, transgender people and prisoners) and their sexual partners [1].

In the last 10 years, new infections showed a 21% increase in Latin America. Despite Brazil having remarkable public policies against HIV, which cover universal access to health care for an average of 80% of people living with it, these percentages about access to antiretroviral therapy (ART) fall to 70%, and only 60% of them reach the suppressed viral load [1].

The ART providing to people living with HIV (PLHIV) and has been the main strategy to control new infections because studies have proved that virus is not transmissible during intercourse when a PLHIV is under ART for at least six months, and under a suppressed viral load at a plasmatic undetectable level [2, 3] making it possible that people maintain or restore their sexual and affective lives after the HIV diagnosis, often conjoining with HIV seronegative partners [4], referred to as HIV serodiscordant or serodifferent couples.

Nevertheless, viral load suppression depends on a variety of factors like knowledge of self HIV serostatus, access to health care assistance, ART adherence, HIV genotype, among others, which could contribute to a failed HIV suppression. In addition, an HIV seronegative partner can become increasingly exposed to the virus, depending on sexual behavior (e.g., receptive intercourse, or ejaculation), the presence of sexually transmitted infections (STIs) [5], or even the challenges related to adherence to medication, stigma and barriers to health assistance [6, 7].

So, in this context, a combined approach to HIV prevention has emerged including behavioral (e.g., consistent condom use), biomedical (e.g., HIV testing and treatment, pre and post-exposure prophylaxis), and structural issues [1]. These combined prevention strategies make it possible to expand risk management among couples living in the HIV context. The diversity of strategies allows greater freedom of choice for the best preventive methods and benefits different marital arrangements considering HIV serodiscordant couples [7, 8]. Therefore, counseling and guidance about sexual HIV prevention for serodiscordant couples is indispensable overall [4, 7].

In Brazil, researchers warn about the invisibility of HIV serodiscordant couples in health interventions. Studies describing serodiscordant partners’ risk, HIV prevalence, as well as their sexual behaviors, are just now emerging [4, 9, 10] and the biomedical management of symptoms and viral control of HIV remains the core of health assistance for PLHIV, whereas it should move beyond the clinic and pivot to a perspective of sexual health that includes their sexual-affective relationships [4, 11] as soon as possible.

In order to contribute to this care perspective, this study aimed to identify HIV serodiscordant couples’ HIV prevalence and determine their associated factors, from a Brazilian city in the State of São Paulo.

## Method

### Study design

This is a quantitative, analytical, cross-sectional study carried out in the five Specialized Care Clinics for PLHIV, in Ribeirão Preto, São Paulo, Brazil, from July 2016 to July 2017.

### Population and sample

To determine the sample size, an expected 62% prevalence in PLHIV with an active sex life, following an HIV diagnosis, was adopted and described from a previous study [12]: a relative error of 10% and significance level of 5%, and a total population of an infinite sample size (*N* = 10.000, for example) was used, where P represents the prevalence of the event of interest, 2 α z represents the level of significance adopted and ε is the relative sampling error. Therefore, the required sample size was *n* = 235.

A non-probabilistic, consecutive-type sample was used, formed by the individuals served in clinics. Ultimately, from 417 PLHIV invited to the study only 20 refused (4.8%), totalizing a sample of 397 PLHIV, 69% larger than what was needed and with a 95% response rate.

Those who agreed to participate in the study and who met the following inclusion criteria were interviewed: they were aware of their HIV diagnosis; had an age equal to or greater than 18 years; were under clinical-outpatient follow-up in at least one of the clinics in the study; and had an active sex life and sexual partnership in the last six months regardless of their partner’s HIV status. Individuals in situations of confinement were excluded, such as: prisoners and those institutionalized and living in support homes.

### Study locations

Brazil has a unified health system that covers universal and free access to health assistance at all levels of care (primary, secondary and tertiary) [13, 14]. Furthermore, the treatment for HIV is available only through this unified health system.

PLHIV outpatient monitoring occurs at the primary and secondary levels of care, in which Specialized Care Services (SCS) team of nursing, infectologists, pharmacists, psychologists, and community health agents helps the client and, the care assistance is supported by national protocols [15].

This study was carried out in the city of Ribeirão Preto, whose health network is spread into five districts facilitating PLHIV to access treatment in their same home micro-region [16]. Consequently, besides treatment, these services offer STI and HIV testing, safe sex counseling, pharmocology and various other services (education, immunization, nursing consultation, etc) as recommended by Brazilian public policies and protocols of HIV care [15].

### Data collection

Data was collected by the researcher and five trained and qualified research assistants. Patient interviews were carried out individually, administered in outpatient settings. The questionnaire (supplementary file) designed for this study was form and content validated by three nurses and a psychologist who are specialists in HIV care and counseling.

### Variables of interest

Sociodemographic data included gender (male or female), age (years), self-reported color skin (white, black, yellow, brown, indigenous), schooling (< 11 years / > 11 years), sexual orientation (heterosexual female, heterosexual male and men who have sex with men) and marital status (single, married, divorced, stable union, widower).

HIV-clinical data included the time span since diagnosis (< 5 years; ≥5 years), a self-report of antiretroviral therapy (ART) adherence in the last six months (yes / no), and plasmatic viral load in the last six months (detectable for values > 50 copies / ml or undetectable for values ≤50 copies / ml). We evaluated all the records of the plasmatic viral load of each participant in the last six months until the interview. We considered as undetectable those who sustained viral load values below 50 copies/ml throughout this period. The opposite was considered detectable.

All the sexual behavior variables were measured considering the past six months until the date of the survey. It included: number and type of sexual partnership (steady, casual, both); HIV status of sexual partners (seropositive or seronegative/unknown), condom use in the last six months (always or inconsistent (no/sometimes)), intercourse with alcohol use (yes/no) or with drugs use (yes/no), talking with the partner about HIV-prevention methods (yes/no), and alcohol and/or drug use by the partner before intercourse (never / sometimes/always).

Health service variables included: receiving HIV sexual transmission guidance from health professionals (yes / no); partner invited to attend the clinic (yes / no); receiving counseling as a couple about HIV sexual transmission (yes / no).

### Data analysis

We applied descriptive statistics to characterize the sample: the absolute (n) and relative (%) frequency, mean, standard deviation (SD), and minimum and maximum n. The Chi-square and Fisher’s Exact tests were performed to analyze associations.

Based on the literature, we assessed the association between all independent variables in our study (socio-demographic, clinical related to HIV infection, behavioral and health service) with the partners’ HIV status (negative/unknown, positive) as the outcome through logistic regression analysis. The “seropositive” category was adopted as a reference category.

The unadjusted and adjusted odds ratios (OR) were calculated from the model parameters with 95% Confidence Intervals (CI) and 5% (α = 0.05) was adopted as the significance level. We used the Statistical Package for the Social Sciences (SPSS) (IBM Corp. Released, 2013) version 22.0 [17] and R (R Core Team, 2018) version 3.5.3 programs [18].

### Ethical aspects

The study was conducted in accordance with the legal and ethical standards of Resolution 466/2012. The study was approved by the Research Ethics Committee of the Ribeirão Preto School of Nursing at the University of São Paulo under decision number 2.369.369.

## Results

The study sample consisted of 397 PLHIV. The prevalence of HIV seronegative/unknown partners was 72.0% (*n* = 286). As described in Table 1, a majority of participants were men (*n* = 257; 64.8%), aged over 35 years (*n* = 280; 70.5%), and have less than 11 years of formal education (*n* = 214; 53.9%).

**Table 1:** Sociodemographic and clinical data of PLHIV and partners HIV-serostatus, Ribeirão Preto - SP, Brazil, 2019; n (%)

Variable	HIV status of Sexual Partner			p^**†**^
	Seronegative/ unknown ***n*** = 286 (72%)	Seropositive ***n*** = 111 (28%)	Total ***n*** = 397 (100%)	
**Age (years)**				
18–24	23 (8.0)	07 (6.3)	30 (7.6)	0.190
25–34	59 (20.6)	28 (25.2)	87 (21.9)	
35–44	100 (35.0)	47 (42.3)	147 (37.0)	
45 +	104 (36.4)	29 (26.1)	133 (33.5)	
**Race**				
White	151 (53.4)	59 (53.2)	210 (53.3)	0.971
Black, mixed, or other	132 (46.6)	52 (46.8)	184 (46.7)	
**Schooling**				
< 11 years	147 (51.4)	67 (60.4)	214 (53.9)	0.108
≥ 11 years	139 (48.6)	44 (39.6)	183 (46.1)	
**Sexual orientation**				
Heterosexual Woman	90 (31.5)	50 (45.0)	140 (35.3)	**0.019**
Heterosexual Man	88 (30.8)	33 (29.7)	121 (30.5)	
MSM*	108 (37.8)	28 (25.2)	136 (34.3)	
**Time since first HIV diagnosis**				
Up to 4.9 years	105 (36.7)	49 (44.1)	154 (38.8)	0.173
≥ 5 years	181 (63.3)	62 (55.9)	243 (61.2)	
**Viral load**				
Detectable	68 (23.8)	41 (36.9)	111 (28.0)	**0.008**
Undetectable	218 (76.2)	70 (63.1)	286 (72.0)	

* Men who have sex with men; ^†^Chi-square test

Heterosexual women showed a relatively larger proportion and number of HIV seropositive partners (*n* = 50; 45%; *p* = 0.019). At the same time, participants with undetectable viral load showed a larger proportion and number of partners with negative or unknown HIV serostatus (*n* = 218; 76.2%; *p* = 0.008).

According to Table 2,most participants who had HIV seronegative/unknown partners (*n* = 286; 72.0%) reported having had sex with only one (62.6%) and steady (52.1%) partner in the last six months and the consistent use of condoms was great among them (*n* = 203; 71.0%; *p* < 0.001). Further, a remarkable number of participants reported the partner never was invited to a clinical appointment (*n* = 219; 76.6%; *p* < 0.001), nor has received counseling as a couple (*n* = 244; 85.3%; *p* = 0.005) from the health team.

**Table 2:** Association between PLHIV sexual behavior and partners’ HIV serostatus, Ribeirão Preto - SP, Brazil. 2019; n (%)

Variables	HIV status of Sexual Partner			
	Seronegative / unknown n = 286 (72%)	Seropositive n = 111 (28%)	Total n = 397 (100%)	p^**†**^
**Number of sexual partners**				
1	179 (62.6)	99 (89.2)	278 (70.0)	**0.001**
2–4	69 (24.1)	10 (9.0)	79 (19.9)	
≥ 5	38 (13.3)	02 (1.8)	40 (10.1)	
**Type of sexual partner**				
Steady	149 (52.1)	106 (95.5)	255 (64.2)	**0.001**
Casual	122 (42.7)	03 (2.7)	125 (31.5)	
Steady and casual	15 (5.2)	02 (1.8)	17 (4.6)	
Alcohol use before sexual relations*				
Yes	126 (44.1)	43 (38.7)	169 (42.6)	0.336
No	160 (55.9)	68 (61.3)	228 (57.4)	
Drug use before sexual relations*				
Yes	59 (20.6)	17 (15.3)	76 (19.1)	0.227
No	227 (79.4)	94 (84.7)	321 (80.9)	
**Conversations with partner about prevention methods**				
Yes	142 (49.7)	69 (62.2)	211 (53.1)	**0.011**
No	144 (50.3)	42 (37.8)	186 (46.9)	
Use of alcohol with partner before sexual relations*				
Sometimes	129 (45.1)	49 (44.1)	178 (44.8)	0.355
Never	143 (50.0)	60 (54.1)	203 (51.1)	
Always	14 (4.9)	02 (1.8)	16 (4.0)	
Use of drugs with partner before sexual relations*				
Sometimes	49 (17.1)	16 (14.4)	65 (16.4)	0.792
Never	230 (80.4)	92 (82.9)	322 (81.1)	
Always	07 (2.5)	03 (2.7)	10 (2.5)	
Condom use*				
Consistent	203 (71.0)	51 (45.9)	254 (64.0)	**0.001**
Inconsistent	83 (29.0)	60 (54.1)	143 (36.0)	
**Received orientation from their health care team**				
Yes	75 (26.3)	63 (56.8)	138 (34.8)	**0.001**
No	211 (73.8)	48 (43.2)	259 (65.2)	
**Partner invited to clinic appointment**				
Yes	67 (23.4)	60 (54.0)	127 (32.0)	**0.001**
No	219 (76.6)	51 (46.0)	270 (68.0)	
**Received couples counseling from the health care team**				
Yes	42 (14.7)	30 (27.0)	72 (18.1)	**0.005**
No	244 (85.3)	81 (73.0)	325 (81.9)	

**†** Chi-Square Test; * in the last six months

Moreover, among those who had HIV seroconcordant partnerships (*n* = 111; 28%), most had only one partner (*n* = 99; 89.2%; *p* = 0.001), steady (*n* = 106; 95.5%; *p* < 0.001); who used to talk about prevention methods (*n* = 69; 62.2%; *p* = 0.011), and referred inconsistently use of condoms (*n* = 60; 54.1%; *p* < 0.001). Although they did not receive counseling as a couple (*n* = 81; 73.0%; *p* = 0.005), they received individual counseling about HIV transmission (*n* = 63; 56.8%; *p* < 0.001) and their partners were invited to a health appointment (*n* = 60; 54.0%; *p* = 0.005).

Table 3 shows the multivariate analysis. PLHIV who had less than 11 years of formal education (0.35 [0.13–0.91] p = 0,031), or had detectable viral load (0.29 [0.12–0.7] *p* = 0,006), or referred inconsistent use of condoms (0.3 [0.13–0.7] *p* = 0,006), were less likely to have had a partner with seronegative/unknown HIV status.

**Table 3:** Factors associated with HIV serodiscordant partnership of people living with HIV. Ribeirão Preto - SP, Brazil, 2019

Variables	ORcrude (CI 95%)	OR Adjusted (CI 95%)	p
**Age**			
18–24	0.87 (0.27–2.79)	0.58 (0.09–3.72)	0.837
25–34	0.73 (0.34–1.58)	0.64 (0.19–2.09)	
35–44	0.78 (0.39–1.56)	0.7 (0.28–1.73)	
≥ 45	1	1	
**Race**			
Not white	1.11 (0.63–1.97)	2.08 (0.95–4.58)	0.065
White	1	1	
**Schooling**			
≥ 11 years	1	1	0.026
< 11 years	0.43 (0.24–0.79)	0.35 (0.13–0.91)	
**Sexual orientation**			
Heterosexual woman	0.67 (0.35–1.29)	0.93 (0.38–2.29)	0.260
MSM*	2.55 (1.12–5.8)	2.58 (0.69–9.66)	
Heterosexual man	1	1	
**Time since HIV diagnosis**			
< 5 years	0.73 (0.41–1.3)	0.55 (0.22–1.34)	0.187
≥ 5 years	1	1	
**Viral load**			
Detectable	0.51 (0.27–0.95)	0.29 (0.12–0.7)	0.005
Undetectable	1	1	
**Number of partners**			
1	1	1	0.371
2–4	2.56 (1.07–6.16)	0.68 (0.17–2.63)	
≥ 5	10.09 (1.31–77.49)	3.75 (0.31–44.87)	
**Type of partner**			
Casual	10.77 (3.22–36.01)	8.12 (1.7–38.8)	0.002
Steady and casual	5.17 (0.63–42.34)	24.8 (1.46–420.83)	
Steady	1	1	
Intercourse under alcohol use**			
Yes	1.19 (0.64–2.21)	0.79 (0.3–2.04)	0.621
No	1	1	
Intercourse under drugs use**			
Yes	1.57 (0.64–3.85)	4.31 (0.96–19.4)	0.049
No	1	1	
**Conversations with partner**			
Yes	1	1	0.117
No	2.12 (1.12–4)	2.11 (0.81–5.44)	
Partner alcohol use before intercourse**			
Sometimes	1.08 (0.59–1.96)	1.07 (0.41–2.83)	0.299
Always	3.7 (0.45–30.51)	14.94 (0.48–469.99)	
Never	1	1	
Partner drugs use before intercourse**			
Sometimes	0.85 (0.36–2.01)	0.16 (0.04–0.76)	0.059
Always	1.06 (0.2–5.64)	0.65 (0.06–7.25)	
Never	1	1	
Inconsistent use of condoms**			
Yes	0.29 (0.16–0.54)	0.3 (0.13–0.7)	0.005
No	1	1	
**Receive orientation from the health care team**			
Yes	1	1	0.001
No	3.68 (2.03–6.68)	5.08 (2.02–12.76)	
**Partner invited to clinical appointments**			
Yes	1	1	0.919
No	3.29 (1.79–6.03)	0.96 (0.4–2.26)	
**Received couples counseling from the health care team**			
Yes	1	1	0.310
No	2.35 (1.23–4.5)	0.62 (0.25–1.57)	

MSM – Men who have sex with men; **in the last six months

The multivariate model draws attention to the likelihood of serodifferent partnerships according to the type of relationship. The chance of being in an HIV serodifferent partnership was 8.12 likely for those who had casual partners (8.12 [1.7–38.8]) and 24.82 times more between those who had steady and casual ones concomitantly (24.82 [1.46–420.83]).

Despite a notable number of participants (*n* = 169; 42.6%) referred to alcohol use before having sex (Table 2), our analysis didn’t identify a significant strength between its use and the HIV serostatus of partners. But those who had sex under drug use were a 4.31 times chance of being in a serodifferent partnership [4.31 (0.96–19.4); *p* = 0.049] (Table 3). Furthermore, surprisingly, the participants who did not receive guidance from the health team were 5.08 times (5.08 [2.02–12.76]) more likely to have an uninfected partner.

## Discussion

Our findings show a high prevalence (72.0%) of PLHIV in a relationship with HIV seronegative/unknown partners, similar to other studies that described representative rates of PLHIV in sexual serodifferent relationships worldwide [2, 3, 8, 19, 20].

In sub-Saharan Africa, for example, around half of couples affected by HIV are in serodifferent relationships [21] and in East and Southern regions these rates reach 80% [22]. In Brazil, there is a lack of official epidemiological and behavioral data on HIV-serodifferent couples in different contexts of the relationship [9], like steady and casual partnerships from Official Epidemiological Data. This fact highlights its invisibility in services, health policies, as well as among social movements and researchers.

Interventions carried out in HIV counseling that encourages joint sexual decision-making among serodifferent couples can improve adherence to effective preventive practices [23]. In this sense, considering the benefits of counseling couples about prevention efforts, it should be integrated into care programs for PLHIV [11, 23, 24].

In our study, the low chance of having an HIV uninfected partner among those who had less than 11 years of study, corroborates other studies which show that there is a higher proportion of PLHIV with more years of education in serodiscordant partnerships [2, 20].

Positively, 72.0% of participants have achieved an undetectable viral load for six months, showing positive efforts to reach viral load suppression whereas they remain in relationships and active sex lives. Further, those who had detectable viral load were less likely to have an HIV-seronegative partner. Such results point to the need to maintain preventive strategies even among HIV seroconcordant sexual partners, considering the risk of transmission of different viral strains, as well as other STIs [2, 25].

The sustained undetectable viral load has a protective effect among couples because HIV is non-transmissible when PLHIV are taking ART and under sustained viral load suppression for at least six months [2, 3]. Nevertheless, the viral suppression is directly linked to ART adherence, and a viral rebound considered as of great risk of HIV transmission occurs within 2 to 3 weeks after stopping treatment [26]. Thus, infectivity and the risk of HIV sexual transmission must be understood within a broader context of behavior challenges [27, 28], and vulnerability since several psychosocial factors interfere in ART adherence and its effectiveness [7, 29].

The use of psychoactive substances (it includes alcohol and drugs), has been associated with suboptimal adherence to ART which leads to a failure of the sustained viral suppression [29, 30]. Therefore, it can affect adherence to preventive strategies among sexual partners.

In our study, participants who used drugs before having sex were likely to have an HIV serodiscordant partnership. Overall, the consumption of these substances is associated with disinhibition, arousal, and activity sexual increase [31]. The concern is that its effects may decrease people’s self-perception of risk or encumber them to communicate or set sex rules, thence favors unsafe intercourse considered at risk for HIV and other STIs [20, 31].

Studies focused on STI transmission, including HIV, have reported more frequently the practice of ‘chemsex’ or ‘Part-n-Play’ among young adults. However, experts in this field suggest that the practice is still underestimated by health professionals [32, 33].

Further, the consistent condom use was quite frequent (64.0%) by our participants, and those who referred inconsistently used it (36.0%) were less likely to be in an HIV serodiscordant relationship. In Brazil, the condom is the cheapest and most easily accessible preventive strategy. It is available for a low price in pharmacies, stores, and supermarkets. Furthermore, it is freely distributed to the population through the unified health system.

The benefits of condom use to prevent HIV sexual transmission are well settled in literature [4, 5, 34]. However, its use is influenced by several factors, like gender relations and, maybe fundamentally, by relations of pleasure, affection, and desire [32, 34, 35]. In addition, the notions of risk and prevention to STI based on condoms are running out as new HIV prevention strategies emerge, being necessary to expand prevention awareness to a combined-strategy perspective beyond the prevalence of condoms [34, 35].

In our findings, there was more PLHIV in casual relationships than in stable ones. Moreover, the regression analysis showed that serodiscordant relationships were 8.1 times more likely among those who had casual partners and 24.8 times more likely for those who had steady and casual ones at the same time.

We hypothesized there are more HIV-negative people than HIV-positive people in terms of prevalence in the city where the study was carried out. So that, it may be a higher chance of having a casual HIV-negative sexual partner. However, there is a lack of studies regarding HIV serodiscordant couples in the region. So, future studies are necessary to a further comprehension of these findings.

Above all, the type of partnership implies how couples negotiate sexual rules, which interferes with the choice and acceptance of strategies for preventing STIs [27, 28, 36]. This is the essential issue surrounding this discussion regarding the type of partnership. Therefore, we highlight that gathering information about sex partnerships enables health professionals to identify the specifics vulnerabilities of each client and thus counsel them about suitable preventive strategies.

Surprisingly, we found out that individuals who did not receive counseling were more likely to have HIV-seronegative partners. Receiving counseling may contribute to facing difficulties concerning stigma, reaching effective preventive strategies among serodiscordant couples [37–39], and supporting the HIV-negative partner to openly express concerns about the risk of HIV trsnamission [37].

This lack in our findings should be interpreted under the light of some circumstances. First, the seropositive partners of participants may have already been under follow-up when they began the relationship. Further, a great number of participants weren’t in a steady relationship.

Beyond that, studies have described health professionals as unprepared to address issues related to sexuality, especially over the unknowledge regarding preventive strategies and resources available for PLHIV and serodiscordant couples [22, 35, 40–42]. One of them identified reluctance from health providers to prescribe or advise preventive strategies even though they are aware of its safety and efficacy are already well established by scientific evidence [40].

The evidently high risk of HIV seroconversion has led serodiscordant couples to difficulties related to sexual practices, which further raises the tensions between worrying about being infected and keeping their sex lives active, often resulting in separation, marital disruption, and decreased sexual activity [22, 38, 43].

Risk management of HIV transmission by serodifferent couples is important not only because of HIV acquisition exposure by the uninfected partner, but also due to the autonomy of choosing the preventive strategies that are convenient for them and to reduce tensions, fear, and anxieties related to the risk of transmission, permiting them to enjoy their sex lives with pleasure and security [22, 35, 43].

For this reason, it is pivotal to welcome PLHIV together with their partnerships in health services and to inform about the several preventive strategies currently available (for example, internal or external condoms, Pre / Post-Exposure Prophylaxis, regular testing for STIs) [22, 38, 42].

It’s necessary to enable these professionals to overcome the stigma, moral judgments and the discomfort in openly discussing sexual behavior [41, 42], so that they further a stigma-free approach, and mediate access to new information on HIV among their clients effectively, adapting to existing singularities, such as different socioeconomic levels or partnership settings.

Finally, we strongly recommend health authorities and managers mobilize educational activities to train and update the knowledge of health providers in order to successfully implement these new approaches.

This study was convenience sampled, therefore it is prone to bias, particularly recall bias that is inherent in cross-sectional surveys. Also, claiming causality between variables is tenuous, since this was cross-sectional. On the other hand, we have more than sufficient participants (69% more than what was needed for robust analysis) and more than one clinic was utilized to insure that facility type would not incur bias.

So, the findings on HIV partner status and relationship types (e.g. steady and casual partners) should be interpreted with caution due to the huge confidence intervals indicating an unstable estimate. Moreover, PLHIV have already been linked to the treatment according to the Brazilian unique health system’s rules, when they agreed to participate in the research. Therefore, there was no interference on these participants’ care linkage. For this reason, this study may not be generalizable to PLHIV who are not in clinical follow-up, nor linked to health services. However, this does not affect the inference on those who are in clinical follow-up and retained to care considering that, in Brazil, the HIV treatment is available only through the public national health system, and it follows the same protocol for patient linkage in the entire national territory.

## Conclusion

Our findings showed a high prevalence of PLHIV who have HIV serodiscordant partnerships. Factors such as having a detectable viral load and inconsistent use of condoms were associated with a lower chance of having an HIV serodifferent partner. Otherwise, those who had casual partners or those who did not receive HIV prevention guidance from health professionals showed a greater chance of having a serodiscordant partnership.

Therefore, we highlight that couples living in the context of HIV require greater visibility by health providers. The health assistance provided daily to PLHIV must move beyond the biomedical management of symptoms and viral control of HIV and pivot to a perspective that respectfully includes the diversity of their sexual partnerships.

## Supplementary Information

**Additional file 1 Supplementary File 1.** Questionnaire of sexual prevention among couples living in the HIV context. This instrument gathers sociodemographic, clinical, sexual, and reproductive questions to evaluate strategies adopted recently to prevent sexual HIV transmission by people living in its context. The original version of this questionnaire is in the Portuguese language. The English translation was carried out to facilitate the study publication after it had finished.

## Data Availability

The data sets used and/or analyzed during the present study are available with the corresponding author upon reasonable request.

## Abbreviations

ART: antiretroviral therapy
CI: Confidence Interval
HIV: human immunodeficiency virus
OR: odds ratio
MSM: Men who have sex with men
PLHIV: people living with HIV
STIs: sexually transmitted infections
SD: standard deviation
SPSS: Statistical Package for the Social Sciences
SCS: Specialized Care Services
UNAIDS: Joint United Nations Program on HIV / AIDS

## References

[1] Joint United Nations Programme on HIV/Aids (UNAIDS). *Unaids Data 2020*. 2020. [Internet] Avaiable in: < https://www.unaids.org/en/resources/documents/2020/unaids-data >.

[2] Rodger AJ, Cambiano V, Bruun T, Vernazza P, Collins S, Degen O (2019). Risk of HIV transmission through condomless sex in serodifferent gay couples with the HIV-positive PARTNER taking suppressive antiretroviral therapy (PARTNER): final results of a multicentre, prospective, observational study. Lancet..

[3] Cohen MS, Gamble T, McCauley M (2020). Prevention of HIV transmission and the HPTN 052 study. Annu Rev Med.

[4] Reis RK, Melo ES, Fernandes NM, Antonini M, Neves LAS, Gir E (2019). Inconsistent condom use between serodifferent sexual partnerships to the human immunodeficiency virus. Rev Latino-Am Enfermagem.

[5] Koff A, Goldberg C, Ogbuagu O (2017). Condomless sex and HIV transmission among serodifferent couples: current evidence and recommendations. Ann Med.

[6] Tadesse WB, Gelagay AA (2019). Risky sexual practice and associated factors among HIV positive adults visiting ART clinics in public hospitals in Addis Ababa city, Ethiopia: a cross sectional study. BMC Public Health.

[7] Yombi JC, Mertes H (2018). Treatment as Prevention for HIV Infection: Current Data, Challenges, and Global Perspectives. AIDS Rev.

[8] Bantigen K, Kitaw L, Negeri H, Kebede M, Wassie A, Bishaw K (2021). Rate of HIV seroconversion among seronegative male partners living with HIV positive women in Addis Ababa, Ethiopia, 2019: A retrospective cohort study. HIV AIDS (Auckl).

[9] Pilcher CD, Bisol CA, Paganella MP, Vallabhaneni S, da Motta LR, Kato SK (2015). Efficient identification of HIV Serodiscordant couples by existing HIV testing programs in South Brazil. PLoS One.

[10] Fernandes NM, Hennington EA, Bernardes JS, Grinsztejn BG (2017). Vulnerabily to HIV infection in serodiscordant couples in Rio de Janeiro. Brazil Cad Saúde publica.

[11] Mashaphu S, Burns JK, Wyatt GE, Vawda NB (2018). Psychosocial and behavioural interventions towards HIV risk reduction for serodiscordant couples in Africa: A systematic review. S Afr J Psychiatry.

[12] Reis RK, Melo ES, Gir E (2016). Factors associated with inconsistent condom use among people living with HIV/Aids. Rev Bras Enferm.

[13] Constitution of the Federative Republic of Brazil: *Constitutional text of October 5, 1988, with the alterations introduced by Constitutional Amendments no. 1/92 through 72/*2013 *and by Revision Constitutional Amendments no. 1/94 through 6/94*. Brazil; 2013. Avaiable in: http://www2.senado.leg.br/bdsf/handle/id/243334

[14] Brasil. Ministério da Saúde. Departamento de Doenças de Condições Crônicas e Infecções Sexualmente Transmissíveis. *Diretrizes para Organização e Funcionamento dos CTA no âmbito da Prevenção Combinada*. Ministério da Saúde, Brasil. 2017. Avaiable in: http://www.aids.gov.br/pt-br/gestores/diretrizes-para-organizacao-e-funcionamento-dos-cta-no-ambito-da-prevencao-combinada .

[15] Brasil. Ministério da Saúde. Departamento de Doenças de Condições Crônicas e Infecções Sexualmente Transmissíveis. *Protocolo Clínico e Diretrizes Terapêuticas para Manejo da Infecção pelo HIV em Adultos*. Ministério da Saúde, Brasil. 2018. Avaiable in: http://www.aids.gov.br/pt-br/pub/2013/protocolo-clinico-e-diretrizes-terapeuticas-para-manejo-da-infeccao-pelo-hiv-em-adultos .

[16] Secretaria Municipal de Saúde de Ribeirão Preto: *Rede Básica*. Ribeirão Preto; 2020. Avaiable in: http://www.saude.ribeiraopreto.sp.gov.br/portal/saude/rede-basica .

[17] IBM Corp. Released 2013. IBM SPSS Statistics for Windows, Version 22.0. Armonk, NY: IBM Corp.

[18] R Core Team. R: A language and environment for statistical computing. R. 2018. Foundation for Statistical Computing, Vienna, Austria. URL https://www.R-project.org/.

[19] Nakku-Joloba E, Pisarski EE, Wyatt MA, Muwonge TR, Asiimwe S, Celum CL (2019). Beyond HIV prevention: everyday life priorities and demand for PrEP among Ugandan HIV serodiscordant couples. J Int AIDS Soc.

[20] Xu JF, Wang PC, Cheng F (2020). Health related behaviors among HIV-infected people who are successfully linked to care: an institutional-based cross sectional study. Infect Dis Poverty.

[21] Wall KM, Kilembe W, Vwalika B, Haddad LB, Lakhi S, Onwubiko U (2017). Sustained effect of couples' HIV counselling and testing on risk reduction among Zambian HIV serodiscordant couples. Sex Transm Infect.

[22] Mwakalapuka A, Mwampagatwa I, Bali T, Mwashambwa M, Kibusi S. Emotional and Relationship Dynamics between HIV SeroDiscordance and Concordance Couples: A Narrative Literature Review and Theoretical Framework. *ARC J Public Health Community Med*. 2017;2(2):1–14. doi: 10.20431/2456- 0596.0202001.

[23] Mthembu J, Hamilton AB, Milburn NG (2020). "It Had a Lot of Cultural Stuff in It": HIV-Serodiscordant African American Couples' Experiences of a Culturally Congruent Sexual Health Intervention. Ethn Dis.

[24] King R, Min J, Birungi J, Nyonyintono M, Muldoon KA, Khanakwa S (2015). Effect of couples counselling on reported HIV risk behaviour among HIV Serodiscordant couples by ART use, HIV status and gender in rural Uganda. PLoS One.

[25] Molla AA, Gelagay AA (2017). Risky sexual practice and associated factors among HIV positive adults attending anti-retroviral treatment clinic at Gondar University referral hospital. Northwest Ethiopia PLoS One.

[26] Hamlyn E, Ewings FM, Porter K, Cooper DA, Tambussi G, Schechter M (2012). Plasma HIV viral rebound following protocol-indicated cessation of ART commenced in primary and chronic HIV infection. PLoS One.

[27] Passaro RC, Castañeda-Huaripata A, Gonzales-Saavedra W, Chavez-Gomez S, Segura ER, Lake JE (2019). Contextualizing condoms: a cross-sectional study mapping intersections of locations of sexual contact, partner type, and substance use as contexts for sexual risk behavior among MSM in Peru. BMC Infect Dis.

[28] Knox J, Boyd A, Matser A, Heijman T, Sandfort T, Davidovich U (2020). Types of group sex and their association with different sexual risk behaviors among HIV-negative men who have sex with men. Arch Sex Behav.

[29] Detsis M, Tsioutis C, Karageorgos SA, Sideroglou T, Hatzakis A, Mylonakis E (2017). Factors associated with HIV testing and HIV treatment adherence: A systematic review. Curr Pharm Des.

[30] González-Álvarez S, Hernández-Huerta D, Madoz-Gúrpide A (2017). Relación entre la adherencia al tratamiento antirretroviral en pacientes VIH+ y el consumo de alcohol, asociado o no al uso de otras sustancias. Adicciones.

[31] Scott-Sheldon LA, Carey KB, Cunningham K (2016). Alcohol use predicts sexual decision-making: a systematic review and meta-analysis of the experimental literature. AIDS Behav.

[32] Souleymanov R, Brennan DJ, Logie C, Allman D, Craig SL, Halkitis PN (2019). Pleasure and HIV biomedical discourse: the structuring of sexual and drug-related risks for gay and bisexual men who party-n-play. Int J Drug Policy.

[33] Hampel B, Kusejko K, Kouyos RD, Böni J, Flepp M, Stöckle M (2019). Chemsex drugs on the rise: a longitudinal analysis of the Swiss HIV cohort study from 2007 to 2017. HIV medicine.

[34] Klassen BJ, Fulcher K, Chown SA (2019). "Condoms are … like public transit. It's something you want everyone else to take": Perceptions and use of condoms among HIV negative gay men in Vancouver, Canada in the era of biomedical and seroadaptive prevention. BMC Public Health.

[35] Irungu EM, Ngure K, Mugwanya KK, Awuor M, Dollah A, Ongolly F (2021). "now that PrEP is reducing the risk of transmission of HIV, why then do you still insist that we use condoms?" the condom quandary among PrEP users and health care providers in Kenya. AIDS Care.

[36] Philpot SP, Bavinton BR, Prestage G, Grierson J, Ellard J, Duncan D (2020). Exploring diversity in HIV research in the sexual partnerships of Australian gay and bisexual men. Arch Sex Behav.

[37] Morton JF, Celum C, Njoroge J, Nakyanzi A, Wakhungu I, Tindimwebwa E (2017). Counseling framework for HIV-serodiscordant couples on the integrated use of antiretroviral therapy and pre-exposure prophylaxis for HIV prevention. J Acquir Immune Defic Syndr.

[38] Larki M, Bahri N, Moghri J, Latifnejad RR (2020). Living with Discordance: A Qualitative Description of the Challenges Faced by HIV Negative Married Women. Int J Community Based Nurs Midwifery.

[39] Musinguzi N, Kidoguchi L, Mugo NR (2020). Adherence to recommendations for ART and targeted PrEP use among HIV serodiscordant couples in East Africa: the “PrEP as a bridge to ART” strategy. BMC Public Health.

[40] Ngure K, Ongolly F, Dolla A (2020). "I just believe there is a risk" understanding of undetectable equals untransmissible (U = U) among health providers and HIV-negative partners in serodiscordant relationships in Kenya. J Int AIDS Soc.

[41] Golub SA, Gamarel KE, Lelutiu-Weinberger C (2017). The importance of sexual history taking for PrEP comprehension among young people of color. AIDS Behav.

[42] Calabrese SK, Mayer KH (2020). Stigma impedes HIV prevention by stifling patient-provider communication about U = U. J Int AIDS Soc.

[43] Tchakounté C, Nkenfou CN, Tchouangueu TF (2020). HIV Serodiscordance among Couples in Cameroon: Effects on Sexual and Reproductive Health. Int J MCH AIDS.

