# Supplementary material for: Serodiscordance predictors among couples in the HIV context: implications for health care
Source: BMC Public Health. 2021 Oct 13;21:1849. doi: 10.1186/s12889-021-11835-0 (PMC8513240; doi:10.1186/s12889-021-11835-0)
Supplement: Supplementary file 1 — Additional file 1 Supplementary File 1. Questionnaire of sexual prevention among couples living in the HIV context. This instrument gathers sociodemographic, clinical, sexual, and reproductive questions to evaluate strategies adopted recently to prevent sexual HIV transmission by people living in its context. The original version of this questionnaire is in the Portuguese language. The English translation was carried out to facilitate the study publication after it had finished. [file 12889_2021_11835_MOESM1_ESM.pdf]

Questionnaire of sexual prevention among couples living in the HIV context

This instrument gathers sociodemographic, clinical, sexual, and reproductive questions to evaluate strategies adopted recently to prevent sexual HIV transmission by people living in its context. We appreciate your participation and ensure keep confidential and anonymous the data provided here.

Inclusion criteria:

Having active sex life and some sexual partnership in the last six months

System number	Interview date:	interviewer
________________________	___ / ___ / ___	______________________

Ribeirão Preto

2016 / 2017

*The original version of this questionnaire is in the Portuguese language. The English translation was carried out to facilitate the study publication after it had finished.

SOCIODEMOGRAPHIC AND CLINICAL DATA

1. Sex assigned at birth?
○Female ○Male

2. Date of birth?
_______/______/______

3. City:
○Ribeirão Preto ○Other __________

4. Schooling:
______________________ years of study

5. Skin color (self-reported)?
○White ○Black ○ Yellow ○Brown ○ Indigenous

6. Marital status:
○ Single (a)○Married / Consensual marriage
○ Divorced ○ widower

7. Do you have kids?
○Yes ○ No

8. If yes, how many kids? ___________________

9. Do you live with a partner?
○Yes ○ No

10. HIV Exposure category
○ Sexual ○ Heterosexual ○ Homosexual ○ Bisexual
○ Blood transfusion ○ Injecting drug use
○ Vertical transmission ○Do not know

11. Current job situation?
○ Public server
○ Formal worker
○ Informal worker
○ Don`t work at moment
○ Domestic worker
○ Retiree

12. Family income monthly? (Gross amount)

__________________________ (Add all income)

13. How many people live in the same house?

____

14. HIV time of diagnosis?
________________________ (months/years)

15. Are you using antiretroviral (ARV)?
○Yes ○No

16. How long have been using antiretroviral therapy (ART)?
_______________________ (months/years)

17. Makes use of other drugs?
○Yes ○No wich are ___________

CHART DATA (Medication / Laboratory Examination)

18. Use of medicines:

21. Chronic diseases:

19. Comorbidities:
○ Diabetes ○ Depression
○ Lipodystrophy ○ Systemic arterial hypertension
○ Dyslipidemia
○ Triglyceridemia
○ Others:______________________

20. Sexually Transmitted Infections in the last 6 months:
○ HPV ○ Trichomoniasis ○ Gonorrhea
○ Syphilis ○ Genital herpes
○ Others:______________________

22. CD4 cells count : _______ mm³

23. Viral load ____________________ copies/mL

24. CD4 Date (__/__/__)

25. Viral Load Date (__/__/__)

26. Type of antirretroviral (ARV):
○ Abacavir ○ Ritonavir
○ Biovir ○ Saquinavir
○ Didanosina ○ Raltegravir
○ Darunavir ○ Leopinavir
○ Atazanavir ○ Indinavir
○ Estavudina ○ Tenofovir
○ Fusamperenavir ○ Enfuvirtida
○ Zidovudina ○ Nevirapina
○ Efavirez ○ Lamivudine

SEXUAL LIFE

Many of the following questions are regarding your sex life and your relationships. Report us if you have any doubts or feel uncomfortable during the interview. Remember that you don't have to answer the question if you prefer to.

27. Type of sexual partnership?
○Steady relationship ○Casual ○Steady relationship and casual

28. Number of partners in the last 6 months? ___________

29. Currently, in general, you have sex with:
○Mens ○Women ○Mens and women ○ Transvestites ○Transsexuals

30. What is the current anti-HIV serology of your partner?
○ Positive ○Negative ○ Unknown

31. Partner in medical follow-up?
○Yes ○No ○Do not Know ○Not applicable

32. If yes, does he(she) uses ART?
○Yes ○No ○Do not know

33. How long are you with your partner?

34. Which kind of sexual practice did you have in the last 6 months?

______________________ (Months/Years)	1. Oral ○<br>2. Vaginal insertive ○<br>3. Vaginal receptive ○<br>4. Anal receptive ○<br>5. Anal insertive ○<br>6. Coitus Interruptus ○

GENITAL TRACT INFECTIONS

35. Did you have any of these clinical manifestations in the last 6 months?<br>○ Genital wounds ○ Genital discharge<br>○ Small genital ulcer ○ Anogenital warts<br>○No ○ Don't know / didn't see<br><br>36. If yes, did you do any treatment?<br><br>○ Yes ○No ○ Not applicable	37. STI history of the current **PARTNER**:<br><br>○ Genital wounds ○ Genital discharge<br>○ Small genital ulcer ○ Anogenital warts<br>○No ○ Don't know / didn't see

HIV TRANSMISSION RISK KNOWLEDGE

38. Which sexual practice has the highest risk of HIV transmission?<br><br>○ Oral ○ Vaginal receptive ○Vaginal insertive<br>○ Anal receptive ○ Anal insertive	40. Do you think that is possible to transmitted HIV to the partner if you are being treated with antiretrovirals and your amount of virus circulating in the body (viral load) is undetectable?<br><br>○Yes ○No ○Do not know
39. Do you know how you can prevent the HIV sexual transmission?<br>○Sim ○Não	41. Does condom use in all sexual relations decrease the risk of HIV transmission?<br>○Yes ○No ○Do not know
42. How do you look for informations about HIV prevention?	48. The low viral load decreases the risk of HIV transmission.

○Television ○Internet ○ Health professionals
○Friends ○Posters/Folderes ○ Campaigns / lectures

○Outhers:_____________________________________

○I don`t look for

43. Do you think that the use and adherence of ART reduce the risk of HIV transmission?
○Yes ○No ○Do not know

44. Do you think have multiple partners increase the risk of HIV transmission?
○Yes ○No ○Do not know

45. “A person living with HIV has little risk of transmitting the virus if they are treated.” Do you agree?

○Agree ○ Disagree ○ Do not know

46. Can a person living with HIV and the partner NOT (discordant couple) transmit HIV to the partner?
○Yes ○No ○Do not know

47. Can a person living with HIV and a partner also (concordant couple) transmit HIV to the partner?

○Yes ○No ○Do not know

○Agree ○ Disagree ○ Do not know

49. The presence of genital ulcers increases the risk of HIV transmission.
○ Agree ○ Disagree ○ Do not know

50. To have sexual intercourse under the influence of alcohol increases the risco of HIV transmission.
○Agree ○ Disagree ○ Do not know

51. Do strategies combined with ARV and condom use decrease the risk of HIV transmission?
○Yes ○No ○Do not know

52. Do you know what is Pre-Sexual exposure prophylaxis (PrEP)?

○Yes ○No

53. Do you know the Post-Sexual Exposure Prophylaxis (PEP)?

○Yes ○No

54. What is the best method of HIV sexual prevention?

○ Male condom
○ Female condom
○ Use ARV to hold undetectable viral load
○ To treat sexually transmitted infections (STIs)
○ To know the partner's HIV serology
○ The combination of these strategies

HEALTH PROFESSIONALS' ACTIONS AND OFFERS

55. Have you ever received HIV sexual transmission information during the counsels by health professionals?

○Yes ○No

56. Which professional?

○ Nursing assistant / technician
○ Nurse ○ Psychologist
○ Social worker ○ Doctor
○ other ____________________

57. Did the health professionals from the service you have been treated talked to you about Post-Sexual Exposure Prophylaxis (PEP)?

○Yes ○No

58. Which professional?

○ Nursing assistant / technician
○ Nurse ○ Psychologist
○ Social worker ○ Doctor
○ Not applicable
○ other ___________________________________

59. In the past 6 months, have you and your partner been seen at the health service as a couple for advice on sexual practices and preventive strategies?

○ Yes ○No

60. By which professional?

○ Nursing assistant / technician
○ Nurse ○ Psychologist
○ Social worker ○ Doctor
○ Not applicable
○ other ___________________________________

61. In the past 6 months, have your partner been invited to go to the health service with you?

○ Yes ○No

62. In the past 12 months, has your partner received guidance on HIV sexual transmission prevention by the health service?

○Yes ○No ○Do not know

63. Have you received orientation about reproductive planning in the HIV context?

○ Yes ○No

64. By which professional?

○ Nursing assistant / technician ○ Psychologist
○ Nurse ○ Social worker
○ Doctor ○ Not applicable
○ other ___________
